# The use of astronomy VLBA campaign MOJAVE for geodesy

**DOI:** 10.1007/s00190-021-01551-3

**Published:** 2021-08-24

**Authors:** Hana Krásná, Leonid Petrov

**Affiliations:** 1 Department of Geodesy and Geoinformation, Technische Universität Wien, Vienna, Austria; 2 Astronomical Institute of the Czech Academy of Sciences, Prague, Czech Republic; 3 NASA Goddard Space Flight Center, Code 61A, Greenbelt, MD USA

**Keywords:** Very Long Baseline Array (VLBA), MOJAVE, Terrestrial reference frame (TRF), Earth orientation parameters (EOP)

## Abstract

We investigated the suitability of the astronomical 15 GHz Very Long Baseline Array (VLBA) observing program MOJAVE-5 for estimation of geodetic parameters, such as station coordinates and Earth orientation parameters. We processed a concurrent dedicated VLBA geodesy program observed at 2.3 GHz and 8.6 GHz starting on September 2016 through July 2020 as reference dataset. We showed that the baseline length repeatability from MOJAVE-5 experiments is only a factor of 1.5 greater than from the dedicated geodetic dataset and still below 1 ppb. The wrms of the difference of estimated Earth orientation parameters with respect to the reference IERS C04 time series are a factor of 1.3 to 1.8 worse. We isolated three major differences between the datasets in terms of their possible impact on the geodetic results, i.e. the scheduling approach, treatment of the ionospheric delay, and selection of target radio sources. We showed that the major factor causing discrepancies in the estimated geodetic parameters is the different scheduling approach of the datasets. We conclude that systematic errors in MOJAVE-5 dataset are low enough for these data to be used as an excellent testbed for further investigations on the radio source structure effects in geodesy and astrometry.

## Introduction

Group delay of an extended source observed with very long baseline interferometry (VLBI) differs from the group delay of a point source. Up to now, the contribution of source structure is not included in routine analysis of VLBI data. It was known for long time that source structure is a significant (e.g., Zeppenfeld 1993; Sovers et al. 2002; Tornatore and Charlot 2007; Shabala et al. 2015; Petrov and Kovalev 2017) or even the major (Anderson and Xu 2018) contributor to the error budget in geodetic VLBI.

One of the most promising ways to compute the source structure contribution to group delay is to generate images from the same VLBI observations, perform their 2D Fourier transform over spatial coordinates, and use it for calculation of structure delay (see, e.g., Petrov and Kovalev 2017). Unfortunately, geodetic observing schedules are not well suited for producing good quality images. A typical geodetic schedule splits the network into a number of ad hoc subarrays, so a subset of stations observes one source and a subset of other stations observes another source at the same time, and upon completion of integration another subset of stations observes the next source. This leads to a substantial reduction of the number of closures in phase and amplitude required for robust imaging. Astronomical schedules usually avoid subarrays. The use of data for geodesy and astrometry from astronomical programs designed for imaging was not common in the past because four to eight intermediate recorded frequencies (IFs) were usually allocated contiguously, while for geodetic applications the frequencies are allocated as wide as possible. As a result, group delay uncertainty at a given signal-to-noise ratio (SNR) was an order of magnitude worse than from geodetic schedules. Although such data were still useful for astrometry (Petrov 2011, 2013), they were too coarse for precise geodesy. A non-contiguous allocation of intermediate frequencies for astronomy projects was rare because usually it was not required, and commonly used software packages, such as AIPS (Greisen 2003), that implemented the fringe fitting procedure do not support direct processing of such data. In case the goal of astronomical observation requires wide spanned bandwidth, e.g., for VLBA (Very Long Baseline Array) Imaging and Polarimetry Survey at 5 GHz (Helmboldt et al. 2007), processing astronomical data in a geodetic/astrometric mode was feasible and provided good results (Petrov and Taylor 2011). However, single-band observations at rather low frequencies such as 5 GHz are affected by the ionospheric refraction, and this limits their usability for geodesy.

Progress in radio astronomy instrumentation resulted in an increase of recorded bandwidth. Since 2016–2020, astronomical observations typically cover frequency bands of 256 or 512 MHz. Group delay precision from these setups is close to the precision reached at geodetic setups. Therefore, the use of astronomical observing program seems feasible as a testbed for studying source structure contribution in detail provided that such a program satisfies two other remaining criteria: a) It observes strong sources, and b) it is conducted at rather high frequencies to minimize the impact of the ionosphere. MOJAVE-5 (Monitoring Of Jets in Active galactic nuclei with VLBA Experiments) suits both these criteria. The program started in 1994 (Lister et al. 1996) and is focused on observations of bright active galactic nuclei (AGNs) with discernible structure at 15 GHz.

## Motivation

Before commencing a thorough investigation of the impact of source structure on astrometry and geodesy results, we need to establish a solid foundation of that work. MOJAVE-5 dataset differs from an usual geodetic dataset a) by the way how it was scheduled; b) by observing frequencies; and c) by the source selection.

An observing schedule consists of a sequence of time intervals called scans when all or a part of antennas of the network record voltage from a given source. Astronomical schedules are usually made by optimization of *uv*-coverage, i.e. projections of the baseline vector on the plane perpendicular to the source direction. The scheduling goal of astronomical experiments is to generate a sequence of observations that covers that plane as uniformly as possible for each program source. Whereas geodetic schedules are usually designed to optimize elevation/azimuth coverage at each station for short time intervals (1–3 h).

Geodetic observations are done at two or more frequencies simultaneously. Since the ionospheric group delay is frequency dependent, multi-band observations allow to derive an ionosphere free combination of group delays. Astronomical observations are usually done at one frequency at once. Therefore, group delay observables from astronomical observations are affected by the ionosphere.

A list of up to 100 objects is usually observed with geodesy schedules. For example, during the year 2020 the mean number of observed sources in the routinely conducted 24 h IVS-R1/R4 (Rapid turnaround sessions of the International VLBI Service for Geodesy & Astrometry) experiments was 57. Sources with extended structures are observed less often than point-like sources. Astronomical schedules have less sources, but they are observed more intensively during an experiment and sources with extended structures are preferably picked.

In Table 1, we summarize the major differences between astronomical and geodetic dataset used in our study. We want to answer the following questions in this paper: (1) What are the metrics of geodetic parameters derived from the MOJAVE-5 dataset? (2) How do these metrics compare to similar geodetic programs? (3) What is the main cause of these differences? And finally, we want to learn whether we can use MOJAVE-5 dataset as a testbed for investigation of the impact of source structure on geodetic and astrometric results. We intend to use MOJAVE-5 dataset in the future to evaluate different methods for computation of source structure contribution to path delay using high-quality images produced by the MOJAVE science team and to evaluate the impact of applying this contribution on estimates of geodetic parameters. The goal of this paper is to evaluate the suitability of the MOJAVE-5 dataset for deriving geodetic parameters and to check whether conclusions derived from processing of astronomical VLBI observations can be generalized to the processing of geodetic observations.

**Table 1:** Overview of major differences between the astronomical dataset MOJAVE-5 bl229 and the geodetic dataset RV&CN

	MOJAVE-5 bl229	RV&CN
Optimization of	*uv*-coverage	Sky coverage
Number of observed frequencies	One	Two
Preferable observed sources	With extended structure	Point-like
Number of sources	Less sources observed intensively	More sources with less observations

## Data analysis

**Fig. 1:**
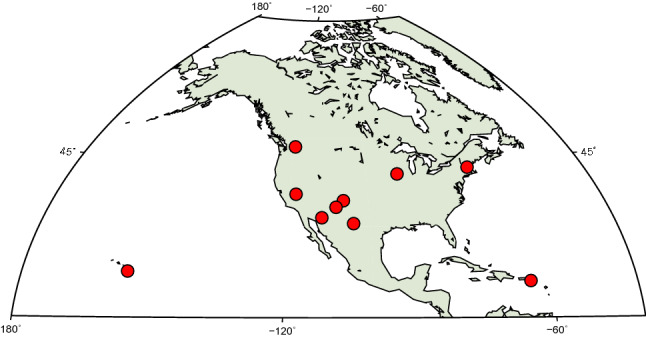
Distribution of the ten VLBA radio telescopes

The VLBA network consists of ten 25-meter radio telescopes located on the US territory (eight in North America, one in the Pacific, and one in the Caribbean), see Fig. 1. The interferometric visibility data of MOJAVE-5 campaign (observing code bl229) at 15.3 GHz (Ku band) with dual circular polarization are publicly available through the National Radio Astronomy Observatory (NRAO) Science Data archive^1^ in the FITS-IDI (Interferometry Data Interchange) format. We processed 33 MOJAVE-5 experiments since September 26, 2016, through July 02, 2020. The first 25 experiments (bl229aa-ay) were observed at independently recorded eight intermediate frequencies of 32 MHz width per polarization using the polyphase filter bank (PFB) operation mode of the digital backend. Since July 2019 (experiment bl229az), the bandwidth of a sub-band has been increased to 64 MHz, containing four sub-bands of 128 channels. MOJAVE-5 campaign has used four IFs of 64 MHz width per polarization using the direct digital converter (DDC) operation mode of the digital backend, also known as DDC personality. In both cases, the total recorded bandwidth per polarization is 256 MHz (see Table 2).

**Table 2:** Lower edge frequency of the sub-bands in the MOJAVE-5 bl229 experiments in GHz

bl229aa–ay	bl229az–bg
15.22400	15.17575
15.25600	15.25575
15.28800	15.31975
15.32000	15.38375
15.35200	
15.38400	
15.41600	
15.44800	

We processed the observations with the fringe-fitting software PIMA (Petrov et al. 2011b) running coarse fringe fitting—bandpass calibration—fine fringe fitting and producing the output databases that include group delays and their uncertainties among other parameters. These quantities serve as input for the data analysis software package pSolve^2^ and VieVS (Böhm et al. 2018).

As a reference dataset, we analyzed 28 geodetic RV (regular geodesy with VLBI) (Petrov et al. 2009) and 6 CN (concurrent with even IVS-R1) dual-band sessions observed at 2.3/8.6 GHz (S/X bands) for the same time span starting with rv119 on September 14, 2016, through July 07, 2020. The RV network consists of the ten VLBA stations plus up to seven other geodetic stations ensuring global geographic coverage. These sessions are designed to provide accurate estimates of the Earth orientation parameters (EOP), a highly accurate terrestrial reference frame (TRF) determination, and source position estimation where the VLBA stations are incorporated into the VLBI reference frame through the inclusion of other geodetic stations with long history of observations. In CN experiments, only the ten VLBA stations participate (Thomas and MacMillan 2020).

To assess the quality of geodetic results, we estimated baseline lengths and EOP and computed their weighted root mean squares (wrms) from the astrophysical MOJAVE-5 program and dedicated geodetic RV&CN experiments. As an extra check, we analyzed the VLBA data in several ways. One solution was produced using the software PIMA for the fringe fitting and pSolve for the analysis. In the second solution, we analyzed group delays produced with PIMA with the geodetic VLBI analysis software VieVS. For the RV&CN experiments, we run another solution with VieVS where we used group delays obtained from Fourfit visibility analysis software (Cappallo 2017). The latter data products in vgosDB format were retrieved from the IVS data archive.^3^ Table 3 contains the parameterization of the solutions in pSolve and VieVS, respectively. Table 4 shows weighted rms of post-fit residuals. The MOJAVE-5 and RV&CN experiments are processed in the same manner with the same parameterization to allow an informative comparison.

**Table 3:** Parameterization of estimated parameters of a single session solution in pSolve and VieVS

*pSolve*	
CRF	fixed to ICRF3 with exception of sources having the $\chi ^2/ndf > 1.5$
TRF	NNT/NNR condition w.r.t. ITRF2014 on VLBA stations with 0.1 mm constraints
ERP	Offset and rate without constraints
Celestial pole offsets	Offset without constraints
Zenith wet delay	B-spline with time span 20 min and sigma of constraints 50 ps/h
Tropo. gradients	B-spline with time span 8 h with sigma of constr. 0.5 mm on offset and 2.00 mm/day on rate
Clocks	B-spline with time span 1 h and constraint sigma 5.e-14 s/s
Baseline clock offsets	Offset with constraint sigma 500 ns
*VieVS*	
CRF	Fixed to ICRF3 with exception of sources identified with pSolve
TRF	NNT/NNR condition w.r.t. ITRF2014 on VLBA stations
ERP	Piecewise linear offsets (pwlo) with time interval 24 h with relative constraints 1 mas
Celestial pole offsets	pwlo with time interval 24 h with relative constraints 0.1 $\mu$as
Zenith wet delay	pwlo with time interval 30 min with relative constraints 50 ps between intervals
Tropo. gradients	pwlo with time interval 3 h with relative constraints 5 mm between intervals
Clocks	pwlo with time interval 1 h with relative constraints 43 ps between intervals, one rate and quadratic term
Baseline clock offsets	Offset without constraints

The statistic value $\chi ^2/ndf$ stands for ratio of the sum of squares of the weighted residuals over the used observations of the specific source to its mathematical expectation

**Fig. 2:**
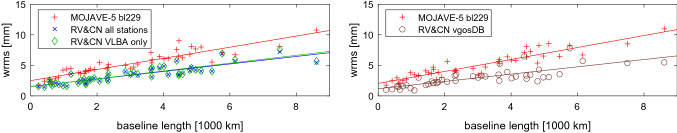
Baseline length repeatability at the VLBA network. Left panel compares baseline scatter computed with pSolve from MOJAVE-5 dataset (red crosses), RV&CN dataset after processing data from all stations (blue x-signs), and RV&CN dataset when non-VLBA observations were dropped (green diamonds). Right panel shows baseline length repeatability computed with VieVS from MOJAVE-5 dataset (red crosses) and RV&CN dataset processed with Fourfit (brown circles)

*Baseline length repeatability.* We ran several solutions, and we compared the scatter in baseline length estimates. We show in Fig. 2 the wrms of the estimated VLBA baseline lengths from solutions computed with pSolve (left panel) and with VieVS (right panel). Red crosses denote the baselines determined from the MOJAVE-5 experiments in both plots. We show in the left plot of Fig. 2 the baseline scatter computed from the RV&CN sessions with the whole scheduled network (blue x-signs) and with observations conducted at the VLBA stations only (green diamonds). The plot demonstrates that dropping the data obtained with non-VLBA stations does not change the wrms of the baseline lengths between the VLBA telescopes significantly. In the whole dataset of RV&CN sessions, 82% of observations was carried out at baselines with VLBA—VLBA antennas. 14% of observations was conducted with one non-VLBA and one VLBA antenna, and 8% of observations was realized with non-VLBA antennas. In both solutions (with and without non-VLBA stations), the NNT/NNR condition was applied to VLBA antennas only. In the right plot of that figure, we compare the MOJAVE-5 bl229 baseline scatter with RV&CN sessions processed with Fourfit. We got approximately the same baseline length scatter from MOJAVE-5 and RV&CN sessions using totally independent software packages. The negligible differences in results from pSolve and VieVS give the confidence that an error in data analysis did not happen.

There is an increase in the baseline length repeatability from a solution using the MOJAVE-5 dataset with respect to the reference RV&CN sessions. The baseline length repeatability differences derived from RV&CN and MOJAVE-5 are about 1.3 mm at a 1000 km long baseline and 3.2 mm at the 8611 km baseline length. The coefficients of the linear regression are summarized in Table 5. We conclude that the baseline length repeatability derived from analysis of single-band 15 GHz MOJAVE-5 experiments is approximately a factor of 1.5 larger than the repeatability derived from the contemporary dual-band 2.3/8.6 GHz geodetic dataset.

**Table 4:** Weighted rms of post-fit residuals in ps

	Min	Max	Median
MOJAVE-5 bl229	11.3	28.7	18.4
RV&CN VLBA only	14.7	37.8	24.1
RV&CN all stations	14.7	40.3	25.2

**Table 5:** Parameters of the fitted linear regression model of baseline length repeatability in the form $a\cdot L + b$ where *L* is length of baseline in mm

Dataset	Software	*a* [ppb]	*b* [mm]
MOJAVE-5 bl229	PIMA, pSolve	0.91	2.50
RV&CN VLBA only	PIMA, pSolve	0.64	1.51
RV&CN all stations	PIMA, pSolve	0.61	1.54
MOJAVE-5 bl229	PIMA, VieVS	0.98	2.04
RV&CN all stations	Fourfit, VieVS	0.60	1.17

**Table 6:** Wrms and median formal error statistics of the estimated EOP from MOJAVE-5 bl229 and RV&CN series

		x-pole [$\mu$as]	y-pole [$\mu$as]	dUT1 [$\mu$s]	dX [$\mu$as]	dY [$\mu$as]
MOJAVE-5 bl229	wrms	228	286	23	169	128
	Median formal error	109	153	9	59	56
RV&CN VLBA only	wrms	126	218	15	89	129
	Median formal error	80	120	6	92	69
RV&CN all stations	wrms	117	130	14	72	87
	Median formal error	57	89	4	86	60

The values for ERP are given w.r.t. IERS 14 C04 time series after trend and bias removal. The celestial pole offsets differences dX and dY were computed with respect to the empirical harmonic expansion heo_20200606.heo

*Earth orientation parameters* The Earth orientation parameters were estimated in a so-called backward solution, i.e. in a solution consistent with globally estimated terrestrial and celestial reference frames from the processed sessions. The orientation and the origin of the TRF are set to have no-net-translation (NNT) and no-net-rotation (NNR) with respect to ITRF2014 (Altamimi et al. 2016) for positions of all ten VLBA stations, and the CRF is oriented by imposing the no-net-rotation condition with respect to ICRF3 (Charlot et al. 2020) coordinates of 26 observed defining sources. We ran several solutions similar to those we introduced in the previous paragraph, and we computed the EOP with software package pSolve. Table 6 shows the wrms of the ERP (polar motion components and dUT1) w.r.t. IERS 14 C04 (Bizouard et al. 2019) time series after a trend and bias removal, whereas the wrms of the celestial pole offsets is given w.r.t. a harmonic expansion heo_20200606.heo produced from analysis of available geodetic VLBI data since 1980 through 2020 using the method presented in Petrov (2007). In addition, the median formal error for all five EOP is summarized in the table. We show three solutions computed with pSolve similar to those introduced by the baseline length repeatability, i.e., EOP from MOJAVE-5 dataset, EOP from RV&CN sessions including all stations, and EOP from RV&CN sessions using the VLBA telescopes only. Estimation of EOP using single band observations at high frequencies was made in the past (e.g., Petrov et al. 2011a). Our recent processing of 37 VLBA experiments at 24 GHz (Krásná et al. 2019) showed that although formal uncertainties were on par with dual-band regular geodetic experiments (60 $\mu$as for x-pole, 80 $\mu$as for y-pole and 5 $\mu$s for UT1), the wrms of the difference with respect to the IERS 14 C04 time series taken as a reference were larger than formal uncertainties by a factor of three for polar motion and a factor of ten for UT1. Table 6 shows that ERP determined from MOJAVE-5 data have the wrms differences with respect to the reference IERS C04 14 by a factor of 1.3 to 1.8 larger than from RV&CN experiments at the same network.

## Differences between MOJAVE-5 bl229 and RV&CN

We recognize there are three major differences between the datasets which may have an impact on geodetic results. First, modeling of the ionospheric path delay was different since MOJAVE-5 was observed at a single band. Second, different scheduling approaches were used due to different goals of the experiments. Third, different radio sources were selected for observations. We isolate these factors and determine which factor has the largest impact on the accuracy of geodetic solutions.

### Ionosphere

The ionosphere is a refractive media. Propagating in the ionosphere, phase delay decreases and group delay $\tau _{gr}$ increases with respect to the ionosphere-free $\tau _{if}$ group delay in the absence of the ionosphere as

1 $$\begin{aligned} \tau _{gr} = \tau _{if} + \kappa {\varDelta \mathrm{TEC}}/f^2_\mathrm{eff} , \end{aligned}$$

where $f_\mathrm{eff}$ is the effective frequency that is within several percent of the recorded central sky frequency, $\varDelta$TEC is the differential Total Electron Content measured in TEC units (TECU, 1 TECU = $10^{16}$ electron/$m^2$):

2 $$\begin{aligned} \varDelta \mathrm{TEC} = \int N_v \, d s_1 - \int N_v \, d s_2 \end{aligned}$$

with $s_1$ and $s_2$ as paths of wave propagation from a source to the first and second station of the radio interferometer, and

3 $$\begin{aligned} \kappa = 10^{-16} \cdot \frac{e^2}{ 2 \, c \, m_e \, \epsilon _o} = 5.308018 \times 10^{10} \; s ^{-1} \end{aligned}$$

where *e*—charge of an electron, $m_e$—mass of an electron, $\epsilon _o$—permittivity of free space, and c—velocity of light in vacuum.

To mitigate the impact of the ionosphere on group delay, geodetic observations are usually conducted at two frequencies simultaneously. Combining group delays $\tau _u$ and $\tau _l$ at the upper and lower frequencies $f_u$ and $f_l$, respectively, we can derive the differential TEC, the ionosphere-free path delay, and the ionospheric contribution in the upper band $\tau _{iu}$ as

4 $$\begin{aligned} \varDelta TEC= & {} \frac{f^{2}_{u} f^{2}_{l}}{f^{2}_{u} - f^{2}_{l}} \quad (\tau _{l} - \tau _{u}), \nonumber \\ \tau _{if}= & {} \frac{f^{2}_{u}}{f^{2}_{u} - f^{2}_{l}} \quad \tau _{u} - \frac{f^{2}_{l}}{f^{2}_{u} - f^{2}_{l}} \, \tau _{l} , \nonumber \\ \tau _{iu}= & {} \frac{f^2_l}{f^{2}_{u} - f^{2}_{l}} \quad (\tau _{l} - \tau _{u}). \end{aligned}$$

Derivations of these equations can be found, for example, in Petrov et al. (2011b). This approach allows to effectively cancel the ionospheric contribution, leaving residual contribution at a level not exceeding several picoseconds (Hawarey et al. 2005).

MOJAVE-5 program used only one frequency. An alternative approach for modeling the ionospheric contribution is to use TEC maps from GNSS observation processing. Applying time and spatial interpolation, we can compute TEC in the vertical direction for each station and each observation. Then, we can relate the TEC in the direction of observation at the elevation angle *E* to the TEC in the vertical direction via a mapping function $M_i(E)$. Considering the ionosphere as a thin shell at height *H*, we can easily derive the ionospheric mapping function as

5 $$\begin{aligned} M_i(E)= & {} \frac{1}{\cos {\beta (E)}},\nonumber \\ \beta (E)= & {} \arcsin \frac{ \cos E }{1 + \frac{H}{R_\oplus }} , \end{aligned}$$

where $R_\oplus$ is the Earth’s radius.

We used Center for Orbit Determination in Europe (CODE) TEC time series (Schaer 1999)^4^ with a resolution of $5^\circ \times 2.5^\circ \times 2^h$. This resolution is relatively coarse and accounts only for a part of the signal. Therefore, our results of processing MOJAVE-5 observations are affected by systematic errors caused by the residual ionosphere.

In order to quantify the residual ionospheric signal, we processed the dual-band RV&CN data set. For the purpose of this study, we consider that the ionospheric-free linear combination of X and S band group delays has no ionospheric contribution. We can form the differences between the ionospheric contribution computed from TEC maps and from X and S band group delays and investigate the properties in terms of a stochastic process.

**Fig. 3:**
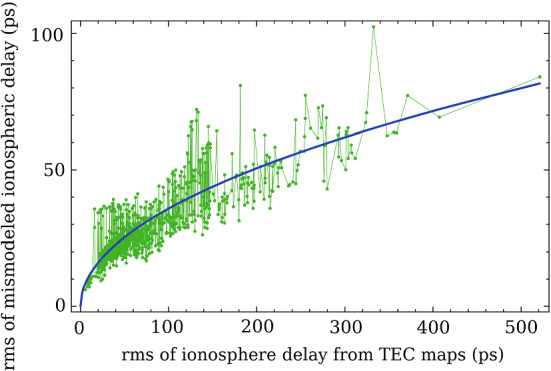
The rms of the errors in the ionospheric path delay as a function of the rms of the variations of the ionospheric group delays derived from TEC maps (green dots). The solid blue line shows a regression in a form of the power law 1/2

Solving for zenith path delays in the neutral atmosphere will pick up a portion of the slowly varying bias, but the ionospheric fluctuations at scales less than several hours will propagate to residuals. However, we can characterize stochastic properties of the residual signal similar to the approaches developed in Petrov et al. (2011b, 2019) and Petrov (2021). The ionospheric path delay fluctuation is a non-stationary process. We can expect that fluctuations at scales *x* will be related to fluctuations at scales *y* via a power law from the general results of the turbulence theory (see Tatarskii 1971). Therefore, we did the following:

First, we computed the mean differences of $d_{gv} = \tau _{ig} - \tau _{iv}$ between the ionospheric path delay at X band computed from TEC maps ($\tau _{ig}$) and from VLBI dual-band observables ($\tau _{iv}$) for every baseline and every experiment in the RV&CN dual-band dataset, and then, we subtracted the mean value from $d_{gv}$. The mean value is the sum of the bias between TEC maps and VLBI ionospheric path delay and a constant instrumental delay in VLBI hardware. Since the constant instrumental delay that may be even larger than the ionospheric signal is not calibrated, the mean value of $d_{gv}$ is meaningless. Then, we computed the rms over $d_{gv}$. We discarded the data with clock jumps that may happen at only one band. We got time series of rms($d_{gv}$), and we examined empirical relationships of rms($d_{gv}$) with other statistics. We found that rms${}^2$($d_{gv}$) has a linear dependence on rms($\tau _{ig}$). The power law dependence between $d_{gv}$ and $\tau _{ig}$ was expected, but the power law coefficient, 2, is purely empirical. Figure 3 demonstrates the time series of $d_{gv}$ and their fit.

We can compute the rms of the ionospheric errors at a given baseline of a given experiment via

6 $$\begin{aligned} \mathrm{rms}(d_{gv}) = \sqrt{ \rho \; \mathrm{rms}(\tau _{ig})}, \end{aligned}$$

where $\rho$ is the empirical coefficient determined from fitting (see Fig. 3) equal to 12.8 ps and the rms is expressed in ps. This empirical relationship allows us to predict the second moment of the residual noise after we perform data reduction for the ionospheric contribution using TEC maps. One can expect that if the TEC variance is greater, the residual errors are also greater. Expression (6) quantifies this dependence.

**Fig. 4:**
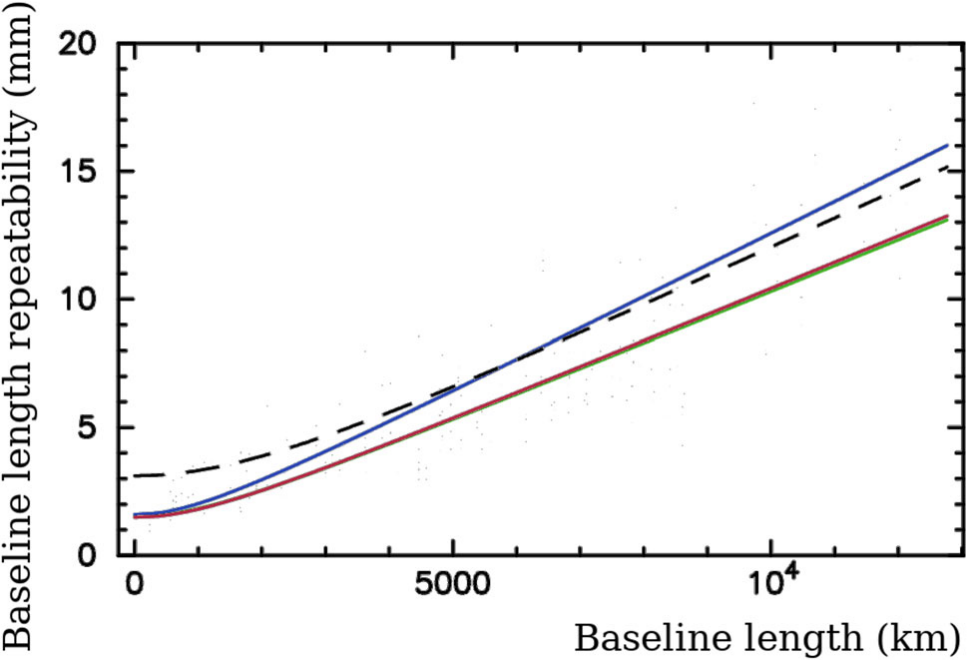
The dependencies of the baseline length repeatability fits on the baseline length. The upper blue curve shows the baseline repeatability for the X band only in the “bx” solution that uses GNSS TEC maps. Two lower very close curves, red and green, show the baseline length repeatability for the “bu” solution that demonstrates the effect of mismodeled ionosphere on Ku band observable, and the reference dual-band solution. The dashed black line shows the baseline length repeatability from the MOJAVE-5 solution

We have computed baseline-dependent additive noise due to mismodeled ionosphere for every baseline and every experiment of MOJAVE-5 program using $\tau _{ig}$. We added that noise to the a priori group delay errors in quadrature and computed new weights. We ran several baseline solutions, computed baseline repeatabilities, and compared them with the reference dual-band solution using RV&CN data. In solution “bx,” we used the ionosphere-free combinations of group delays, added the contribution of the ionosphere $\tau _{iu}$ to them, and processed these data the same way as MOJAVE-5 data, i.e., performing data reduction for the ionosphere using CODE TEC maps and inflating a priori group delay uncertainties for the additional noise due to mismodeling the ionosphere. In the second solution “bu,” we simulated how the deficiency of CODE TEC model would have impacted our RV&CN solution, as if these experiments ran at 15.3 GHz instead of 2.3/8.6 GHz. To achieve this, we re-scaled $\tau _{iu}$ by the square of the frequency ratio $(8.64/15.28)^2 \approx 0.32$. Figure 4 shows a fit in the form $\sqrt{(a\,L)^2 + b^2}$ for all these solutions. The baseline length repeatability from MOJAVE-5 solution is shown by the dashed line.

We found that the impact of the mismodeled ionosphere on the baseline length repeatability of VLBA data collected in 2016–2020 at 15.3 GHz during Solar minimum is negligible. Therefore, an increase in the baseline length repeatability from a geodetic solution using the MOJAVE-5 dataset with respect to the reference dual-band RV&CN solution cannot be explained by the unaccounted contribution of the ionosphere. This result should not be extrapolated to other estimated parameter, such as source position, and should not be extrapolated to epochs of the Solar maximum.

**Fig. 5:**
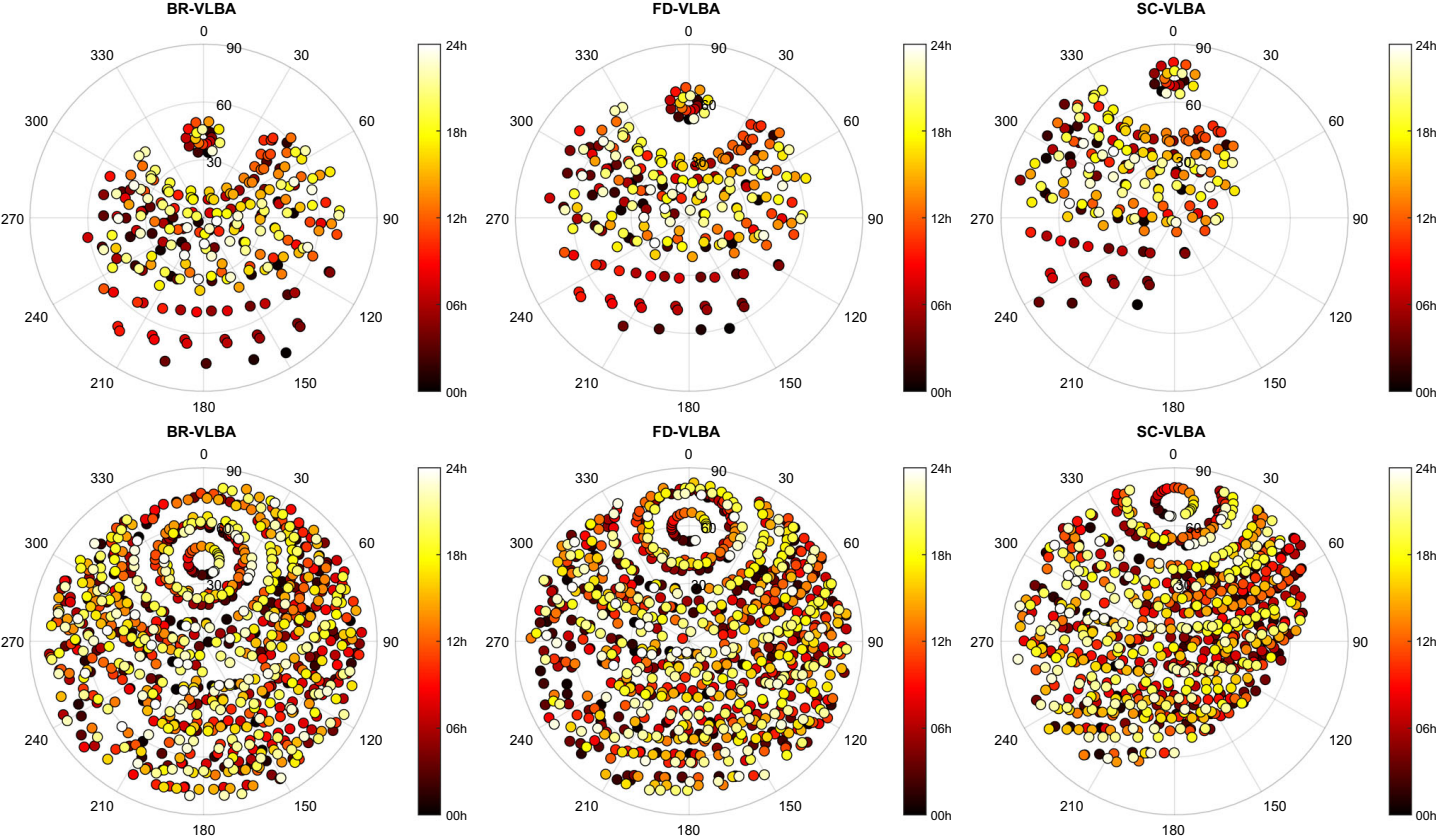
Sky coverage at three VLBA stations: BR-VLBA, FD-VLBA, and SC-VLBA during the bl229bc MOJAVE-5 experiment (upper plots) and the cn1924 experiment (lower plots)

### Scheduling

Scheduling of a VLBI experiment is a complex task. The scheduler has to evaluate several criteria which lead to the best schedule according to the focus of the current experiment.

The major criteria for design of the geodetic schedule are the sky coverage over the individual stations, number of observations, and scan duration. The general scheduling concept of the established software sked for the geodetic sessions can be found in Gipson (2010). Evenly distributed observations over all elevation angles at a given station ensure a good decorrelation of station dependent parameters such as station height, zenith wet delay, clock parameters, or baseline clock offsets (e.g., Nothnagel et al. 2002), and therefore, such schedule can be regarded as station-centric. A large number of observations in general improves the accuracy of the estimated geodetic parameters due to higher redundancy. The challenge for the scheduling software is to find the best compromise between a) the long antenna slew time needed for the best sky coverage in a short interval (1–3 h) allowing a high time resolution of the estimated parameters and b) the short antenna slew time allowing for a high number of observations with sufficient scan duration and high signal-to-noise ratio.

Astronomic VLBI schedules are mainly scheduled using Sched (Walker 2020). The primary goal of the MOJAVE-5 bl229 experiments is to provide best images of jets in active galactic nuclei. Therefore, their schedule is optimized to track a small set of sources (30) in a 24-h session in ten scans per source for a total on-source time of $\sim 35$ minutes. A given source is scheduled to have at least six antennas providing observations of larger than $10^\circ$ elevation.^5^ Such schedule is considered as source-centric.

**Fig. 6:**
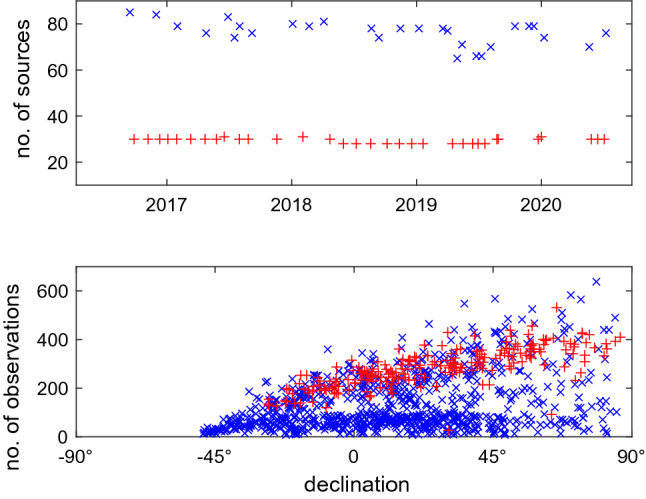
The upper plot shows the number of observed sources in each session. The lower plot depicts the median number of observations for each source. The red crosses stand for the bl229 experiments and blue x-signs for the RV&CN experiments

In Fig. 5, we show the sky coverage during a 24-h observing session at three selected telescopes (BR-VLBA, FD-VLBA, and SC-VLBA) where colors depict the time passed since the session start. As an example, we show the sky coverage during the MOJAVE-5 session bl229bc observed on December 22, 2019, in the upper plots and the cn1924 session observed with the same network on December 09, 2019, in the lower plots. Table 7 summarizes the average number of scans in a 24-h experiment at each of the ten VLBA telescopes computed over the investigated time period (September 2016–July 2020). We see that about twice the number of scans at each telescope was observed in the geodetic experiment with shorter integration time than in MOJAVE-5 observing sessions that used longer integrations. Figure 6 presents the total number of observed sources in each session (upper plot) and the median number of observations during a 24-h session for each source computed over the respective four-year period. The median number of observed radio sources is 30 in MOJAVE-5 sessions, and 78 in RV&CN sessions. Comparison of the number of observations for each source during a whole session shows that 95% of the AGNs observed in MOJAVE-5 sessions have more than 150 observations, whereas only 35% of the sources were observed in RV&CN that often. Our results confirm that geodetic schedules are designed to provide a good sky coverage for each station and the sources are picked up to improve the azimuth/elevation coverage regardless of how often they are observed in a given experiment.

**Table 7:** Mean number of scans at VLBA telescopes in one session computed over the period of interest (September 2016–July 2020)

	Br	Fd	Hn	Kp	La	Mk	Nl	Ov	Pt	Sc
MOJAVE-5 bl229 series	245	245	241	248	251	204	251	252	235	219
Geodetic RV&CN experiments	451	485	445	493	483	357	467	487	451	423

### Simulations

To touch the effect of source structure, we run simulations of the observations. Using the simulation VieVS tool Vie_SIM (Pany et al. 2011), we replaced group delay observables with synthetic artificial group delays provided by the random noise generator as

7 $$\begin{aligned} \tau _{gr} = \tau _{mod} \,\,\, + \,\,\, (\tau _{clk} + \tau _{zwd} + \tau _{fl}). \end{aligned}$$

We added three stochastic error sources to the theoretically computed time delay ($$\tau _{mod}$$): delay caused by the turbulence in the troposphere ($$\tau _{zwd}$$), station clock ($$\tau _{clk}$$), and the Gaussian noise ($$\tau _{fl}$$) with $$\sigma $$=20 ps that accounts for the thermal noise and instrumental errors. We used the model of Nilsson et al. (2007) for simulation of zenith wet delay implemented in VieVS. In the framework of that approach, we considered that the atmospheric turbulence for every station is described with a structure function with the refractive index structure constant $$C_n = 1.8 \times 10^{-7} \; m ^{-1/3}$$, the effective height $$H = 2~km $$, and the constant wind velocity $$v =$$ 8 m/s toward east. For the simulations, we computed the covariance matrix between group delays for each pair of observations of a given station and used them for computation of the full weight matrices under an assumption that the atmospheric turbulence is a stationary process. The simulation of station clocks was performed with an Allan standard deviation of $$1\cdot 10^{-14}$$ at 50 min. We did not include modeling source structure into simulation.

**Fig. 7:**
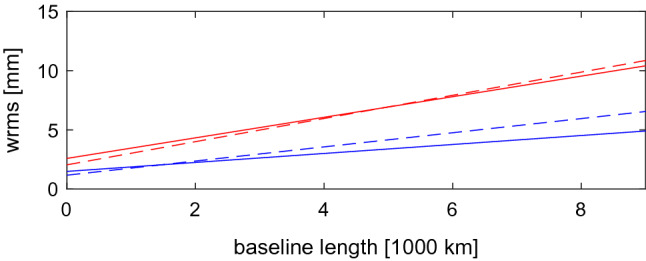
The wrms of baseline length from real (dashed line) and simulated observations (solid line). The upper red lines show the baseline length repeatability from analysis and simulation of MOJAVE-5 data. The low blue lines show results of analysis and simulation of RV&CN data

We have computed baseline length repeatabilities from simulated RV&CN and MOJAVE-5 datasets. The regression lines in a form $a\cdot L + b$ for simulated and real data are shown in Fig. 7. We see from these plots that simulation results show even larger disparity in repeatabilities between MOJAVE-5 and RV&CN data than we saw in real observations. We should note that $\tau _{clk}$ and $\tau _{fl}$ in equation (7) are exactly the same for both datasets, and $\tau _{zwd}$ that is dependent on elevations and time is similar in both datasets because it was derived from the same model. Therefore, the stochastic model we used for MOJAVE-5 and RV&CN simulations is essentially the same. This finding pinpoints the origin of discrepancies in the results obtained by processing real data: differences in schedules.

**Fig. 8:**
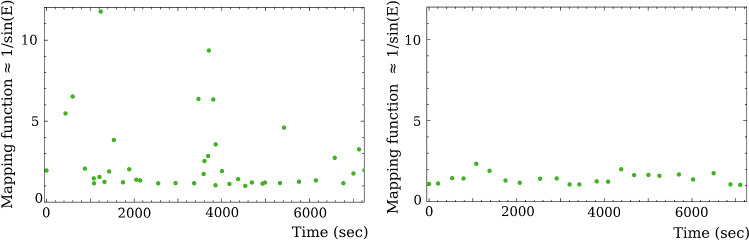
The distribution of observations over the mapping function for station LA-VLBA within first two hours of an experiment. Since deviation of the mapping function from 1/sin(elevation) is small at elevations above 10 deg, mapping function 1/sin(elevation) is used here for illustrative purpose. *Left: * geodetic experiment rv119. *Right: * astronomical experiment bl229aa

Since atmospheric path delays and clock functions are modeled in a form of an expansion over the B-spline basis with a time span of 20–60 minutes, in order to decorrelate these two groups of nuisance parameters and the station vertical component, observations at significantly different elevations are required. Figure 8 shows that the spread of observations over mapping function (approximately reciprocal to sine of elevation angle which equals the partial derivative of the time delay w.r.t. zenith wet delay) for the geodetic experiment rv119 is noticeably wider and observations at elevations below $30^\circ$, which corresponds to mapping function $>2$, appear more often than in the astronomical experiment bl229aa.

**Table 8:** The wrms of the simulated EOP from MOJAVE-5 bl229 and RV&CN experiments observed with the VLBA network. The values are given w.r.t. the IERS 14 C04 time series after trend and bias removal

	x-pole	y-pole	dUT1	dX	dY
	[$\mu$as]	[$\mu$as]	[$\mu$s]	[$\mu$as]	[$\mu$as]
MOJAVE-5 bl229	158	225	16	82	98
RV&CN VLBA only	118	184	10	82	97

In order to look at the problem in more detail, we investigated correlations between estimates of the vertical site position and atmospheric path delays. From the real data, we computed the median correlation coefficient − 0.22 for RV&CN sessions and − 0.35 for MOJAVE-5 sessions. Since these numbers depend on the elevation angles of the observations, we considered the cosine of the median elevation angle as a factor. The median elevation angle over all sessions in the dataset is $33^\circ$ for RV&CN and $47^\circ$ for MOJAVE-5. Therefore, the multiplication of the median correlation coefficient with this factor brought the reduced median correlation coefficients closer together, i.e. − 0.18 for RV&CN sessions and -0.24 for MOJAVE-5. We ran a series of solutions using RV&CN data and flagged out observations below a certain elevation angle. Table 9 summarizes the findings. An increase in the elevation cutoff results in an increase in the baseline length repeatability. MOJAVE-5 has few observations below elevations $30^\circ$ and none below $20^\circ$. The achieved baseline length repeatability from MOJAVE-5 experiments is similar to the repeatability from RV&CN experiments when observations below 20–$25^\circ$ are not included in a solution.

**Fig. 9:**
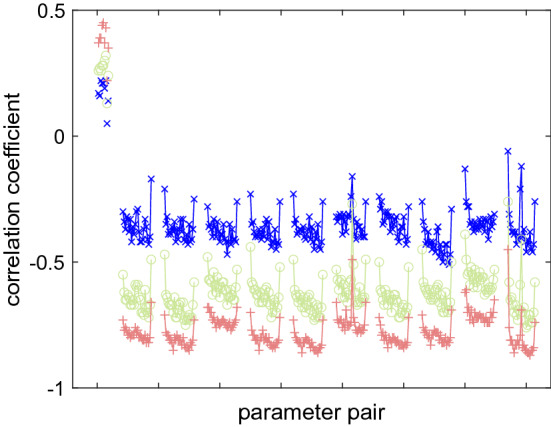
Correlation coefficients in simulated geodetic experiment cn1924 for cutoff elevation 3$^\circ$ (blue x-signs), 20$^\circ$ (green circles), and 30$^\circ$ (pink crosses). The order of parameter pairs at the x-axis: (1st–9th): station’s vertical component and clock offset for all stations except of the clock reference; (10th–last): station’s vertical component and a residual atmospheric zenith path delay (estimated as pwlo every hour) for all ten stations (the solid line connects coefficients that belong to the same station)

Figure 9 shows individual correlation coefficients in simulated geodetic experiment cn1924 for cutoff elevation 3$^\circ$, 20$^\circ$, and 30$^\circ$. The median correlation coefficient between vertical displacement and clock offset at the respective station is 0.19, 0.27, and 0.39 for the increasing cutoff elevation angle. The median correlation coefficient between vertical component and a residual atmospheric zenith path delay is − 0.37, − 0.64, and − 0.79 when the elevation cutoff is increasing. This proves that the strategy including radio sources under low elevations in the schedule over short periods of time allows to decorrelate station dependent parameters in the data analysis and to provide better baseline length repeatability.

We also investigated the residual EOP estimates from the simulated RV&CN and MOJAVE-5 datasets with respect to the IERS C04 14 time series taken as a reference. The results of this simulation are presented in Table 8. The simulation results confirm about the same disparity of 20-60% of the wrms from MOJAVE-5 and RV&CN dataset as in the results derived from real observations (compare with Table 6).

**Table 9:** The coefficients of the baseline length repeatability regression in a form of $a \cdot L + b$ as a function of elevation angle from processing RV&CN VLBA observations

Elevation cutoff	*a* [ppb]	*b* [mm]
$0^\circ$	0.55	1.46
$5^\circ$	0.55	1.46
$10^\circ$	0.70	1.35
$15^\circ$	0.90	1.14
$20^\circ$	1.09	1.14
$25^\circ$	1.22	1.14
$30^\circ$	1.86	1.49

**Fig. 10:**
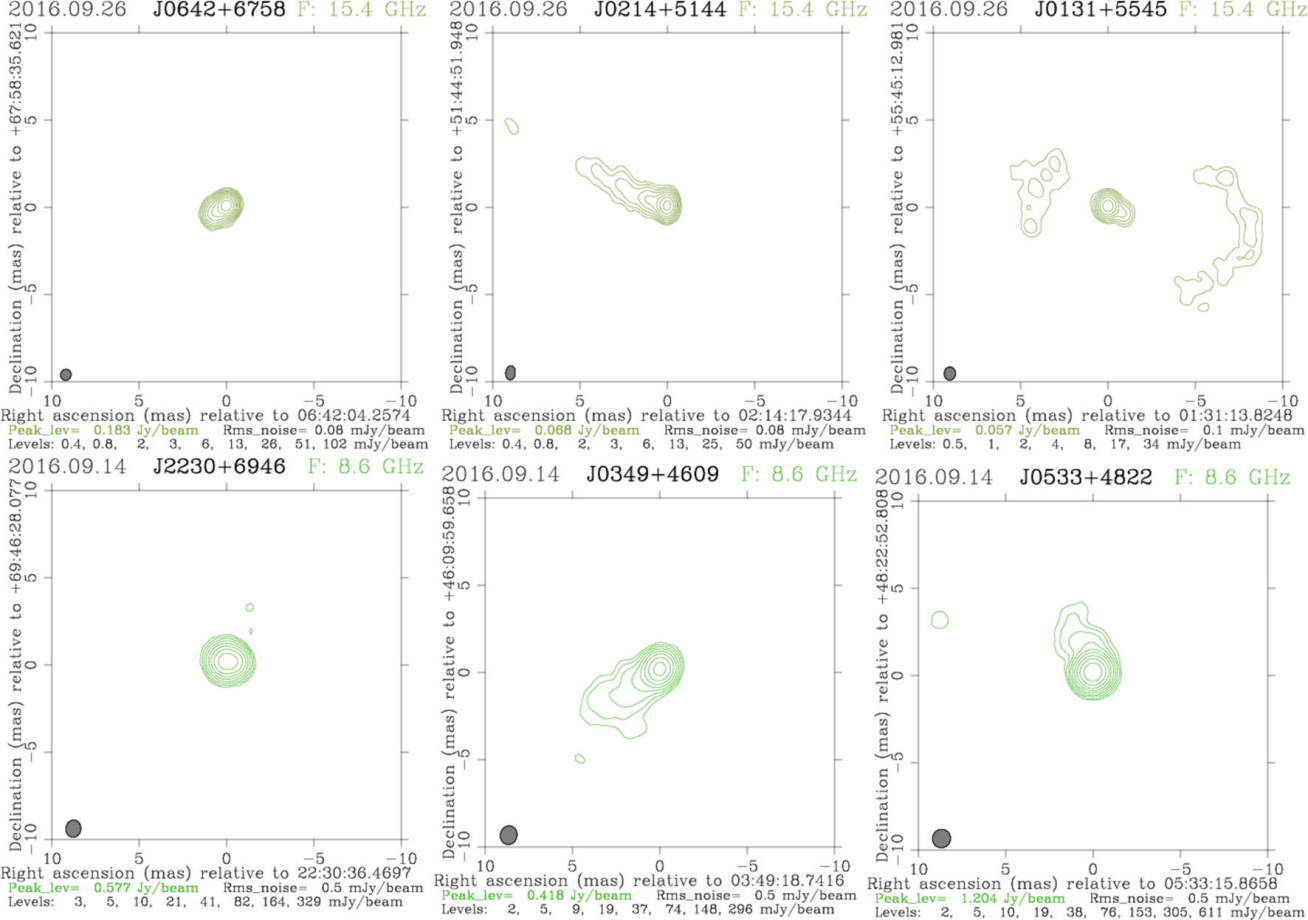
Images of three most observed sources (0636 + 680 with SI 2, 0210 + 515 with SI 3, and 0128 + 554 with SI 4) in the bl229aa MOJAVE-5 experiment (upper plots) and in the rv119 experiment at X band (lower plots: 2229 + 695 with SI 2, 0345 + 460 with SI 2, 0529 + 483 with SI 2). We have produced images from rv119 ourselves. The images in FITS format are available in the Astrogeo VLBI FITS image database http://asteogeo.org/vlbi_images. Information about the structure index for the X band sources was taken from the Bordeaux VLBI Image Database available at http://bvid.astrophy.u-bordeaux.fr

### Insight into a possible contribution of the source structure to the baseline length

The first indicator of astrometric source quality based on source structure corrections was developed by Fey and Charlot (1997). They derived the so-called structure index (SI) from VLBI source images as the median value of the group structure delay ($\tau _{str}$) determined for pixels in a $512 \times 512$ *uv*-grid for all baselines shorter than the diameter of the Earth. Figure 10 shows images of the three most observed sources in the first session of each dataset. In the session bl229aa, these sources are 0636 + 680, 0210 + 515, and 0128 + 554; in the session rv119, the most observed sources are 2229 + 695, 0345 + 460, and 0529 + 483. We computed the SI for all sources observed in bl229aa. For that calculation, we used maps provided by MOJAVE team^6^ and split the sources in the four SI groups according to the median value of calculated structure delay corrections. Among 30 sources observed in bl229aa, 3 sources have SI 1, 14 sources SI 2, 8 sources SI 3, and 5 sources have the highest SI 4.

**Fig. 11:**
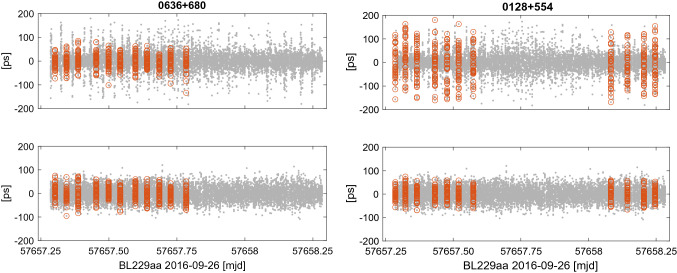
Post-fit residuals in session bl229aa for real (upper plots) and simulated (lower plots) observations. Highlighted are sources 0636 + 680 with SI 2 on the left and 0128 + 554 with SI 4 on the right

We compared the post-fit residuals in session bl229aa from real observations $v_{real}$ (upper plots) and from simulated observations $v_{sim}$ (lower plots) as shown in Fig. 11. As an example, we highlighted the most observed source 0636 + 680 in this session which has structure index 2 and 0128 + 554 with structure index 4. The comparison shows that the scatter of delay residuals for 0636 + 680 is similar to real and simulated observations and the rms reaches 29.6 ps and 29.3 ps, respectively. The rms of delay residuals of source 0128 + 554 with extended structure is 2.5 times larger when computed from real observations compared to simulated ones, 72.3 ps and 27.4 ps, respectively. We computed the rms of delay residuals for every source in the bl229aa experiment and built the difference between the rms from real and simulated observations. The median value of the rms difference was calculated as

8 $$\begin{aligned} \varDelta \mathrm{rms_{med}} = \mathrm{med (rms}(v_{real}) \; - \; \mathrm{rms}(v_{sim})) \end{aligned}$$

over each source group with respect to the structure index. The obtained median values are summarized in Table 10. We see that the difference between simulated and real delay residuals is raising with an increasing source structure index since the structure group delay is not modeled in the simulated observations. With the SI 1 taken as reference, the rms of the delay residuals increases by about 36 ps for sources with SI 4. The lower wrms of post-fit residuals from processing real observations compared to the simulated ones for sources with low structure indices (SI 1 and SI 2) is manifested by the negative $\varDelta \mathrm{rms_{med}}$. It is due to the fact that the random Gaussian noise with the rms of 20 ps that had been added to simulated path delay in our simulation is too high for these compact sources.

We see that the source structure contribution increases the rms of the post-fit residuals, but such an increase even for a subset of sources with strong radio jets picked up for an astronomical program does not have a noticeable impact on baseline length repeatability. Source structure causes not only random but also systematic errors, but their impact on the baseline length repeatability is insignificant. We exercise a caution to generalize this result to source position estimates. This requires a further investigation that is beyond the scope of present work.

**Table 10:** Median of rms differences $\varDelta \mathrm{rms_{med}}$ between delay residuals from real and simulated observations in bl229aa. Sources are divided in four groups according to their structure index, i.e. according to their median group structure delay $\mathrm{med} (\tau _{str})$. $N_\mathrm{sou}$ stands for number of sources in the group

SI	$\mathrm{med} (\tau _{str})$ [ps]	$N_\mathrm{sou}$	$\varDelta \mathrm{rms_{med}}$ [ps]
1	0–3	3	− 11.3
2	3–10	14	− 6.6
3	10–30	8	9.0
4	30–$\infty$	5	25.0

## Conclusions

We have processed 33 diurnal astronomical observing VLBI sessions at 15 GHz under program MOJAVE-5 and 34 diurnal VLBI geodetic dual-band observing sessions at 2 and 8 GHz under programs RV and CN. Both observing sessions ran at the same ten-station VLBA network with baseline lengths in a range from 237 to 8612 km at approximately the same time interval 2016.7–2020.5.

We found that while the median wrms of post-fit residuals from MOJAVE-5 program was lower than from RV&CN, 18.4 ps versus 24.1 ps, important metrics of the geodetic quality of solutions, such as baseline length repeatability and wrms of the differences of the ERP with respect to the reference IERS C04 times series, were a factor 1.3 to 1.8 worse. We investigated the origin of these discrepancies. We have established that modeling the ionospheric path delay using the GNSS TEC maps was adequate for processing 15 GHz data during the Solar minimum, and the errors of these TEC maps did not affect baseline length repeatability at a noticeable level. We investigated whether the source structure can be a factor, since MOJAVE-5 targeted objects with strong radio jets and we have not found evidence it affected baseline length repeatability. Finally, we ran solutions with simulated right hand sides for both MOJAVE-5 and RV&CN programs. The stochastic model used for these simulations was almost the same. We were able to reproduce discrepancies in baseline lengths and EOP time series statistics.

We have established that the major factor that causes discrepancies in baseline length repeatability is a more agile schedule of RV&CN experiments that includes more scans at low and high elevations at short time intervals 1–3 h than astronomical experiments. We showed that the correlation coefficients between the station vertical component and atmospheric zenith path delay increase with an increasing elevation cutoff angle. When we removed observations below 20–$25^\circ$ elevations in RV&CN, we got a similar repeatability as in MOJAVE-5 program.

Although the use of single-band astronomical VLBI data from MOJAVE-5 program for geodesy provided less accurate results than the use of VLBI data from the dedicated geodesy RV&CN campaign, the baseline length repeatability is still below 1 ppb. This gives us a good estimate of the impact of remaining systematic errors that are specific for MOJAVE-5. This very low level of systematic errors confirms that MOJAVE-5 dataset is an excellent testbed for investigation of the effect of source structure on astrometry and geodesy in full detail.

## Data Availability

All observation data were retrieved from publicly available database https://archive.nrao.edu/archive. The TEC time series was downloaded from ftp://ftp.aiub.unibe.ch/CODE. Software packages which are necessary to reproduce the results are open access and can be downloaded from http://astrogeo.org and https://github.com/TUW-VieVS/.

## Footnotes

1 https://archive.nrao.edu/archive.

2 http://astrogeo.org/psolve.

3 Available at https://ivscc.gsfc.nasa.gov/products-data.

4 Available at ftp://ftp.aiub.unibe.ch/CODE.

5 http://www.physics.purdue.edu/astro/MOJAVE/data.html.

6 Available at https://www.physics.purdue.edu/MOJAVE.
